# Effect of Longer Pre-Starter Diet Allowance on Post-Weaning Performance of Lightweight Piglets

**DOI:** 10.3390/ani14233471

**Published:** 2024-12-01

**Authors:** Francesc González-Solé, David Solà-Oriol, Sandra Villagómez Estrada, Ramon Muns, José Francisco Pérez

**Affiliations:** 1 Animal Nutrition and Welfare Service (SNiBA), Department of Animal and Food Science, Autonomous University of Barcelona, 08193 Bellaterra, Spain; david.sola@uab.cat (D.S.-O.); sandra.villagomez@ute.edu.ec (S.V.E.); josefrancisco.perez@uab.cat (J.F.P.); 2 Faculty of Veterinary Medicine and Agronomy, Veterinary Medicine Department, Universidad UTE, Quito 17012764, Ecuador; 3 Agri-Food and Biosciences Institute, Large Park, Hillsborough, Co Down, Northern Ireland BT 26 6DR, UK; ramon.muns@afbini.gov.uk

**Keywords:** weaned pigs, mortality, target body weight

## Abstract

First-phase diets are formulated to help pigs reach a target body weight before switching to a more affordable diet. However, these transitions typically take place on a fixed schedule, which may disadvantage low-body-weight piglets due to their underdeveloped digestive systems and limited feed intake capacity. This study examined the effects of offering low-body-weight (BW) pigs a pre-starter diet until they reached a target BW on growth performance and BW variability. A total of 528 pigs ([Landrace × Yorkshire] × Pietrain), weaned at day 21, representing the smallest 50% of their batch, were divided into medium (5.35 ± 0.31 kg) and small (3.97 ± 0.21 kg) groups based on weaning BW. Pigs received a pre-starter diet either for 10 days (FIXED) or until they reached a target BW of 7.9 kg (TBW), followed by a starter feed. Growth performance between the TBW and fixed strategies was similar over the 36-day period. Overall, mortality was higher in small pigs, though small pigs fed using the fixed strategy showed higher mortality than the rest of the groups between days 10 and 36. This study concludes that offering a pre-starter diet based on target BW, instead of a fixed time, did not significantly improve growth performance or batch uniformity but reduced mortality in small pigs from days 10 to 36.

## Simple Summary

This study evaluated the effects of providing weaning pigs with a pre-starter diet until they reach a target body weight (7.9 kg) before transitioning to a more affordable diet, compared to offering it until a fixed day (10 days post-weaning). Pigs were categorized as medium or small based on their weaning body weight. The results demonstrated that extending the pre-starter diet until a target body weight was achieved did not affect overall growth nor body weight variability but did reduce mortality in small pigs between days 10 and 36. Given that this study was conducted without zinc oxide and with lower protein levels than previous studies, the findings highlight the need to reassess nutritional strategies for improving small pigs’ growth and reducing body weight variability. Future research should explore the potential impact of setting a target body weight in the allowance of a starter diet, as well as assess its long-term effects.

## 1. Introduction

The variation in growth rates among pigs in commercial herds can have detrimental economic consequences for producers [1]. Thus, the implementation of strategies to mitigate its impact is necessary. Although approaches such as sorting pigs by weight after weaning have gained popularity among farmers, it has been demonstrated that this practice only reduces variation within a pen at the time of sorting and does not have a carry-over effect unless individualized treatments across different groups are implemented [2].

The implementation of phase feeding after weaning is crucial to minimize growth checks at weaning and to maximize feed intake and growth performance during the nursery period and subsequent stages [3]. Phase-feeding systems involve subsequent compound diets, each with lower nutrient specifications and costs than the previous one and each formulated to meet the requirements of the average pig [4]. However, diet transitions usually occur on predetermined days, without considering the body weight (BW) variability within the batch.

Under these circumstances, low-BW pigs at weaning, which usually have a poorer developed digestive tract compared to their average counterparts [5], are often introduced to less digestible and cheaper diets before their gastrointestinal tract is properly developed or mature. This situation might lead to an escalation in the influx of undigested nutrients, including protein, into the distal segments of their gastrointestinal tract, the microbial fermentation of which can have a detrimental effect on gut health [6]. This may delay their adaptation to post-weaning diets and can negatively impact their performance [7], further increasing BW variability within a batch.

Previous studies have already determined that providing increased amounts of a standard first-phase dietary regimen at weaning has positive effects on pigs’ growth performance [3,8,9,10,11,12,13]. Similarly, in growing–finishing pigs, providing a fixed allowance of the first two diets to all pens, rather than changing diets on an age basis, allowed the lightest pigs to partially catch up to their larger counterparts in slaughter weight [14]. However, except for one study [13], the rest of the studies implemented their dietary treatments following either a fixed allowance or time regime, without considering that smaller pigs may have higher requirements for a particular diet. Hence, this study proposes a strategy that involves allowing pigs to consume a pre-starter diet until they reach a target BW, rather than offering a limited amount of the pre-starter diet or offering it until a fixed date. It is hypothesized that this approach will have a positive impact on piglets’ performance and contribute to reduced batch-weight variability. Therefore, the objective of this study was to investigate the effects of providing weaned pigs with a higher allowance of a pre-starter diet until they reached a target BW, on their growth performance and the BW variability of the batch.

## 2. Materials and Methods

### 2.1. Animals, Housing and Diets

**Figure 1:**
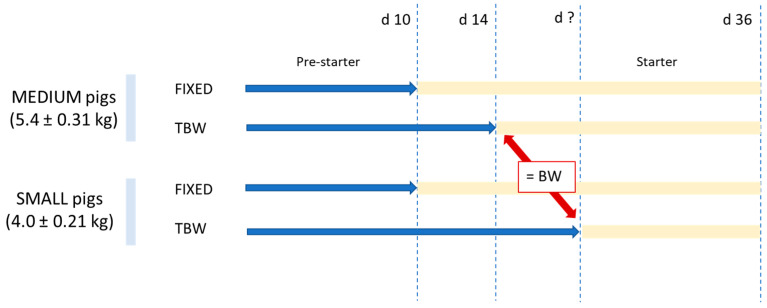
Schematic representation of the experimental design of the study.

This trial was conducted in the nursery unit of a commercial farm in Catalonia (northeast of Spain) throughout the nursery period (d 1 to 36 post-weaning). A total of 528 male and female weaned commercial pigs ([Landrace × Yorkshire] × Pietrain), weaned at d 21, were moved to the nursery unit to be used in the trial. These pigs represented the smallest 50% of their batch (BW of 4.7 ± 0.74 kg). Pigs had access to creep feed during lactation. Each nursery pen (3 m^2^ in floor area) had a commercial non-lidded hopper (TR5, Rotecna, Spain) and a nipple drinker to ensure ad libitum feeding and free access to water. At weaning, animals were individually ear-tagged and classified into two blocks according to initial BW: medium pigs—5.4 ± 0.31 kg; and small pigs—4.0 ± 0.21 kg. Each block contained 24 pens of 11 mixed-sex pigs. Ten days after weaning, pens were assigned to two experimental feeding strategies (12 replicates for each strategy). Half of the pigs within the medium and small BW categories were switched to the starter diet that same day (d 10) following a fixed-day approach (FIXED). The remaining pigs were allowed to consume a pre-starter diet until they reached a specific BW, following a target-BW approach (TBW). The target BW was established as the average BW of the medium pigs in the TBW strategy at d 14 (Figure 1).

All pens were offered the same pre-starter and starter diets, with experimental groups differing only in feeding strategy. The pre-starter diet was formulated to contain 2544 kcal of net energy (NE)/kg, 18.5% crude protein (CP) and 1.39% SID Lys (Table 1) to meet the nutritional requirements of newly weaned pigs (5–7 kg of BW) [15]. The starter diet was formulated to contain 2458 kcal of NE/kg, 17.89% CP and 1.25% SID Lys (Table 1) to meet the nutritional requirements of nursery pigs (7–12 kg) [15]. No antimicrobials nor therapeutic doses of zinc oxide (ZnO) were used in the experimental diets.

Pigs were individually weighed at weaning (d 0), at d 10 and at d 36. Additionally, pigs within each BW category were weighed at the time of the diet change for the TBW group. A subset of small pigs in the TBW group was weighed at d17 to estimate when they would reach the target BW.

Feed disappearance from each pen/hopper was measured throughout the experimental period. The average daily feed intake (ADFI), average daily gain (ADG) and feed conversion ratio (FCR) were calculated for the experimental period. The average daily intake of energy and lysine was determined using the ADFI and the formulated values for each diet. The conversion efficiency of energy and lysine was calculated by dividing the intake of energy or lysine by the weight gain over the specified period.

### 2.2. Statistical Analysis

Data analysis was conducted using the open-source software R v4.0.3 (R Foundation for Statistical Computing, Vienna, Austria). To compare growth performance between the two feeding strategies, separate T-tests were conducted for each BW category. Furthermore, the ADG, ADFI and FCR of all groups during the period from d 0 to 36 were analyzed using a two-way ANOVA in a factorial arrangement. Mortality data during the period from d 0 to 10, from d 10 to 36 and during the whole period were analyzed as counts of deaths using a Poisson regression model, with the number of pigs per pen serving as the weighting factor in a factorial arrangement. The models included the fixed effects of feeding strategy and of BW category, and their interaction. The significance level for determining statistical significance was set at α = 0.05, while tendencies were assessed at α = 0.10. Pairwise comparisons between groups were explored for the variables that showed a significant interaction.

## 3. Results

At d 14, the medium pigs fed using the TBW strategy reached 7.90 ± 0.36 kg, while the small pigs fed using the TBW strategy reached a similar weight (7.84 ± 0.36 kg) by d 21. Therefore, the diet of the small pigs fed using the TBW strategy was changed at d 21.

On average, pigs fed using the fixed strategy consumed a total of 1.78 ± 0.254 kg/pig (medium) and 1.66 ± 0.261 kg/pig (small) of the pre-starter diet. Meanwhile, pigs in the TBW group consumed a total of 2.96 ± 0.254 kg/pig (medium) and 4.89 ± 0.754 kg/pig (small) of the pre-starter diet. Therefore, the medium and small pigs in the TBW group consumed 1.18 kg and 3.23 kg more pre-starter diet (*p* < 0.001) than piglets in the fixed group, respectively (see Table S1).

**Figure 2:**
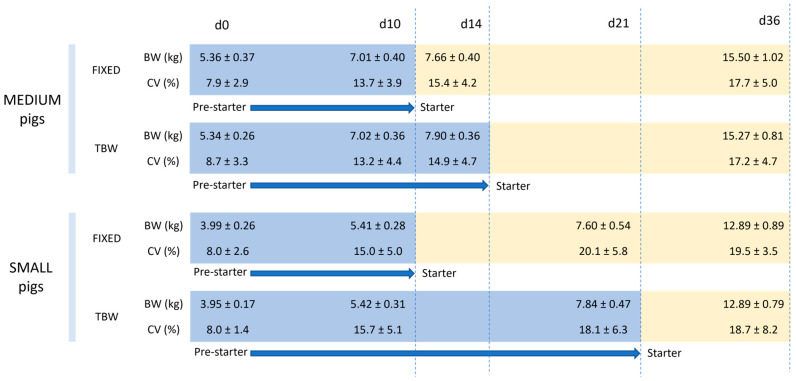
Effect of feeding strategy on the evolution of the body weight (BW) and coefficient of variance (CV) of small (weaning weight—4.0 ± 0.21 kg) and medium (weaning weight—5.4 ± 0.31 kg) pigs for two different feeding management strategies. Values are expressed as mean ± standard deviation. Differences in BW and CV between both strategies were non-significant (*p* > 0.05). FIXED: feeding strategy consisting of allowing pigs to consume a pre-starter diet until d10 post-weaning; TBW: feeding strategy consisting of allowing pigs to consume a pre-starter diet until achieving 7.9 kg of BW.

The BW and CV within each pen throughout the experimental period are represented in Figure 2. No significant differences in the BW or CV within each pen were observed between the fixed and TBW groups during the experimental period.

The ADG, ADFI and FCR calculations for each specific period are detailed in Table 2 and for the entire experimental period (d 0–36) are detailed in Table 3. From d 10 to 14, medium pigs fed using the TBW strategy exhibited a significantly higher ADG (*p* = 0.001) and ADFI (*p* = 0.029) and showed a tendency towards a decreased FCR (*p* = 0.069) compared to medium pigs fed using the fixed strategy. Similarly, from d 10 to 21, small pigs fed using the TBW strategy showed a tendency towards a higher ADFI (*p* = 0.097) than small pigs fed using the fixed strategy. However, when the pigs fed using the TBW strategy were offered the starter diet (medium: days 14–36; small: days 21–26), they showed numerically lower ADG and ADFI values than pigs fed using the fixed strategy, resulting in comparable growth performance for the entire experimental period (days 0–36). Mortality was higher in small pigs throughout the entire study period (*p* < 0.001) and demonstrated a significant interaction between feeding strategy and BW category between days 10 and 36 (*p* = 0.007; Table 3). Specifically, small pigs fed using the TBW strategy exhibited higher mortality during this period compared to the other groups.

From the pig feed consumption data, energy and lysine daily intake as well as their conversion efficiency were calculated (Table 4). Medium pigs fed using the TBW strategy had a superior energy and lysine intake than those fed using the fixed strategy from days 10 to 14 (*p* < 0.05). Likewise, their energy and lysine conversion efficiency tended to be better during the same period (*p* < 0.10). On the other hand, in the small pigs group, from days 10 to 21, the pigs fed using the TBW strategy consumed a higher energy and lysine content (*p* < 0.05) than the pigs fed using the fixed strategy, and their conversion efficiency was numerically worse, although no significant differences were detected (*p* > 0.10).

## 4. Discussion

This study aimed to explore the potential benefits of extending the pre-starter diet allowance in young (weaned at 21d) and small pigs (smallest 50% of a typical weaner batch) until they reached a specific BW, compared to implementing a fixed-day diet change. Contrary to the initial hypothesis, the present study did not find an improvement in growth performance when pigs received an extended pre-starter diet allowance (TBW—e.g., until they reached a 7.9 kg BW) compared to when the diet was changed at a set day (FIXED—e.g., d 10 post-weaning). A diet change at d 10 led to a temporary decrease in feed intake and growth for pigs fed using the fixed strategy. Pigs fed using the TBW strategy also experienced slight growth retardation after the diet change, although it occurred at a higher BW and, arguably, maturational stage. Consequently, both groups of pigs ended the nursery period with similar BW. Additionally, small pigs exhibited a higher mortality rate compared to medium pigs throughout the study, while no effect of feeding strategy was observed for the overall period. However, between days 10 and 36, after implementing the differential dietary approach, small pigs fed using the TBW strategy showed a mortality rate comparable to that of medium pigs and lower than that of small pigs fed using the fixed strategy. This finding suggests that extending the pre-starter diet allowance might improve survival rates among the smallest pigs during the nursery period.

The absence of a growth improvement in the TBW group contradicts most previous studies that reported positive effects on growth or feed efficiency when pigs were offered a higher allowance of first-phase diets [3,4,8,9,10,12,13,16]. The exception is one study [17], which also did not observe performance improvements. However, it should be noted that the weaning weight and age in the present study (4.66 ± 0.74 kg and 21 days of age) were considerably lower than those in previous studies that examined a similar feeding strategy (>7 kg average BW and >28 days of age at weaning). This difference can be partly attributed to the selection of the smallest 50% of pigs from their weaning batch for this trial. Younger and smaller pigs are more vulnerable to the challenges of weaning and typically exhibit lower feed consumption, which likely influenced the results [18]. Therefore, comparisons must be approached with caution due to this crucial difference, and more studies should be performed taking into consideration different weaning scenarios. Nevertheless, it is worth mentioning that the present study differed from previous research in additional aspects that could have contributed to the divergent outcomes. Firstly, most of these previous studies offered an extra 6 kg [3,8,9,10] or 4 kg [12] of first-phase diets. Meanwhile, in this study, the difference in the amount of pre-starter diet consumed between the two strategies was relatively lower—1.18 kg for medium pigs and 3.23 kg for small pigs. Therefore, it could be possible that the extra amount of pre-starter diet consumed by pigs in the TBW strategy, particularly the medium pigs, may not have been sufficient to cause a discernible effect on growth performance. However, Douglas et al. (2014) [4] reported that providing 3 extra kg of a first-phase diet benefited the growth of pigs of a light BW at birth. Nevertheless, this effect was only observed when combined with a higher nutrient specification diet that provided an extra quantity of nutrients than the standard requirements for weaned pigs. Additionally, Huting et al. (2019) [16] observed a weight × feeding regime interaction effect when they provided an additional 2.7 kg of first-phase diet. Specifically, they observed that light pigs who consumed a larger quantity of first-phase diets were 1.2 kg heavier than the light pigs in the control group. However, this effect was not observed in heavy pigs, indicating that smaller pigs may particularly benefit from this feeding strategy, probably due to improved early digestive development which led to improved growth performance. It is important to note, though, that this was a long-term effect, observed at 7 weeks post-weaning, when the pigs had already reached a body weight of approximately 35 kg. This timeframe falls beyond the scope of the present study. However, Huting et al. (2018) [19] found no significant benefit of feeding a post-weaning diet enriched with essential amino acids (20% higher than the standard diet) in the growth of lightweight piglets. Despite expectations that additional amino acids could help offset their low initial weight, these piglets did not achieve better final weights or growth rates compared to those on the standard diet.

In addition, some of the studies that observed significant differences in growth performance employed a strategy that involves providing a higher quantity of the first two post-weaning diets, which are formulated with a high nutritional value, before transitioning to a third-phase diet (grower or weaner diet) with lower energy, protein and lysine content [8,9,10,12]. In these studies, the differences in growth became noticeable towards the end of the nursery period or even in the long term, after the pigs had already commenced consumption of the grower diet [8,9,10,12]. For example, Magowan et al. (2011) [8] compared the effects of offering 2 kg and 4 kg of the first- and second-phase diets, respectively, versus 4 kg and 8 kg, to pigs weaned at an approximately 9 kg BW. Pigs fed 4 kg and 8 kg of the diets showed reduced ADFI and improved FCR when they reached a BW of around 16 kg. However, differences in BW were not observed until the pigs reached an approximately 30 kg BW. Similarly, Muns et al. (2018) [9] compared the effects of offering 2 kg and 6 kg of the first- and second-phase diets, respectively, to pigs weaned at a 9.5 kg BW, versus offering 6 kg of both the first- and second-phase diets. Improvements in ADG and FCR were observed within the first 3 weeks post-weaning, but differences in BW did not reach statistical significance until the 16th week after weaning. In contrast, the TBW strategy implemented in the current trial only extended the provision of the pre-starter diet, without introducing a distinct starter diet with potentially significant differences in nutritional composition.

Thus, it could be argued that we might have observed more differences between strategies in our study if the nutritional value of the starter diet had been significantly poorer than that of the pre-starter diet. Additionally, it could be hypothesized that including a target BW for the allowance of a starter diet has potential as a strategy to reduce the variability within a production batch.

Another difference to consider is that most of the studies cited above were conducted before the ban on the use of ZnO as an antimicrobial in post-weaning diets, and, therefore, their experimental diets included ZnO at high doses. In contrast, our study did not include ZnO at therapeutic doses. Zinc, primarily supplemented as ZnO, is well known for its antimicrobial properties, which can reduce diarrhea incidence, improve intestinal morphology, and modulate genes related to intestinal inflammation, ultimately contributing to reduced post-weaning mortality [20].

In the absence of therapeutic ZnO, our diets were formulated with a lower CP content to minimize the risk of gastrointestinal disturbances. The pre-starter and starter diets contained 18.5% and 17.9% CP, respectively, in contrast to studies conducted prior to the zinc ban, which utilized higher CP levels ranging from 23.3% to 20% in the first two post-weaning diets [3,8,9,10,12,13,16,17,21]. The reduction in CP levels has shown to reduce fecal score and the expression of proinflammatory genes, although it might also reduce pig’s performance after weaning [22]. Although essential SID amino acids were balanced to meet the recommendations for weaned pigs [15], the lower CP content may have limited the pigs’ full growth potential and, as a result, influenced the results. For example, there are suggestions that extra supplementation of individual amino acids, such as arginine and glutamine, may enhance the growth of lightweight piglets [23].

Despite pigs in the TBW group showing a higher energy and lysine intake from the time of the pre-starter diet onwards, their efficiency of utilization was only improved in the medium pigs between days 10 and 14. For small pigs, energy and lysine efficiency of utilization was numerically worse between days 10 to 21. Feed energy and lysine conversion efficiency refers to the effectiveness with which pigs convert the nutrients present in their feed into body protein. The main reason for this lack of nutrient efficiency in small pigs could be the immaturity of their digestive system, which impairs the ability of small pigs to utilize nutrients effectively [7]. Since zinc plays a critical role in structural development and intestinal physiology (including digestion and nutrient absorption), it is plausible that small pigs in our study had experienced slower maturation compared to those traditionally supplemented with extra doses of zinc. This poor nutrient utilization is also related to the additional energy expenditure required to maintain body temperature, boost the immune system and manage high stress levels, which are likely naturally occurring challenges in pigs with lower body weight [24]. Consequently, less energy is available to support growth. Hawe et al. (2020) [13] also reported that providing diets until a target BW was achieved during the nursery period did not improve the efficiency of energy and lysine utilization. However, their study found a sustained improvement in feed intake, leading to increased BW gain. In contrast, the pigs in the TBW group of the current trial lost their feed intake advantage as soon as they were switched to the starter diet.

Although there was no effect on growth performance, extending the allowance of the pre-starter diet led to a reduction in mortality rates in small pigs between d 10 and 36. This positive effect could be linked to the superior quality of the pre-starter diet compared to the starter diet. The pre-starter diet included ingredients like sweet milk whey, acid whey, porcine digestible peptides (PDP) and spray-dried plasma (SDP), which are commonly added to enhance palatability and improve diet digestibility. In contrast, the starter diet replaced these ingredients with higher levels of cereal and soybean products. Including these highly digestible ingredients in the pre-starter diet supports piglets’ adaptation to post-weaning diets, as their digestive capacity is initially limited [7]. In this study, however, this difference did not translate into improved nutrient utilization efficiency in small piglets fed using the TBW strategy. It may, however, have reduced the flow of undigested nutrients to the distal part of the intestinal tract, where fermentation of undigested material can lead to intestinal inflammation, a key factor in post-weaning diarrhea [6]. In addition to its enhanced digestibility, the pre-starter diet contained biologically active ingredients such as SDP and PDP, which have demonstrated immune-modulating properties [25,26]. The specific combination of 2% PDP + 1% SDP used in the pre-starter diet has been shown to positively influence gene expression related to intestinal function, particularly in maintaining barrier integrity and supporting immune response [27].

Consistent with our findings, Leliveld et al. (2013) [17] reported a numerical reduction in mortality among pigs provided with 3–4 kg of phase 1 diet and 9–12 kg of phase 2 diet, compared to those fed 1–2 kg of phase 1 diet and 3–6 kg of phase 2 diet. In their study, both phase 1 and phase 2 diets also included high-digestibility milk-derived products, which were removed in phase 3. This is the only study to test varying allowances of first-phase diets without observing significant changes in growth performance. Similarly, Berrocoso et al. (2012) [28] did not find an effect of diet complexity on growth rate in pigs post-weaning but observed a benefit on fecal score using this strategy. The rest of the studies testing first-phase diet allowance mentioned above observed improvements in growth performance but did not report any effect of the diet strategy on mortality rate. This discrepancy might be explained by the higher vulnerability of pigs in the present trial compared to those in earlier studies. Young, small pigs might not benefit from higher nutritional composition but might derive greater advantage from strategies that support their survival, especially in the absence of therapeutic doses of ZnO in the diet supporting piglets’ health. However, it is important to note that the difference in mortality rates between feeding strategies in small pigs may have influenced the growth performance results. The higher mortality rate in pigs fed using the fixed strategy may have selectively removed slower-growing individuals, resulting in an artificially higher average growth rate compared to what would have been observed if those pigs had survived.

The results from our study, conducted without ZnO in the diets and with subsequent lower levels of CP, suggest the need to reassess nutritional strategies to improve the growth of small pigs and/or reduce weight variability. Nonetheless, our study only followed pigs until d 36 post-weaning. Further work using current post-weaning requirements (i.e., without ZnO and lower levels of CP) and following pigs up to slaughter are needed.

## 5. Conclusions

In conclusion, the strategy of extending the pre-starter diet allowance, formulated without ZnO and with low CP levels (18.5%), until weaning pigs reached a target body weight had no significant effect on growth performance, body weight uniformity or the efficiency of energy and lysine conversion of small pigs. However, this strategy may have contributed to improved survival rates among the smallest pigs during the nursery period. Future studies should investigate the potential impact of incorporating a target body weight into starter diet allowances and examining the long-term effects of this strategy.

## Figures and Tables

**Table 1:** Composition of the experimental diets; % as-fed basis.

Item	Diets	
	Pre-Starter	Starter
Ingredients, %		
Corn	34.3	50.8
Broken rice	11.0	5.0
Wheat	6.0	10.0
Barley	3.0	7.0
Wheat bran	2.5	1.0
Soybean meal 47	10.0	12.0
Extruded soybeans	6.3	2.6
Soybean protein concentrate	3.3	6.6
Sweet milk whey	9.0	-
Lactose	3.5	-
Acid whey	2.2	-
Porcine digestible peptides 62% CP	2.0	-
Spray-dried plasma 80% CP	1.0	-
Sucrose	1.7	-
Lard	1.0	0.5
Mono calcium phosphate	0.56	1.06
Calcium carbonate	-	0.56
Salt	0.16	0.53
Vitamin–mineral premix ^1^	0.40	0.40
L-Lysine liquid 50%	1.04	1.01
DL-Methionine	0.34	0.28
L-Threonine	0.31	0.29
L-Valine	0.25	0.20
L-Isoleucine	0.10	0.05
L-Tryptophan	0.13	0.10
Calculated composition		
Dry Matter, %	89.5	87.8
Net Energy (NE), kcal/kg	2544	2458
Ash, %	4.5	4.5
Crude Protein, %	18.5	17.9
Ether Extract, %	4.2	3.7
Starch, %	35.3	45.2
Neutral Detergent Fiber, %	7.03	8.80
Calcium, %	0.33	0.50
Total P, %	0.53	0.58
Digestible P, %	0.30	0.30
Na, %	0.35	0.22
Cl, %	0.36	0.36
SID amino acids		
Lys, %	1.390	1.250
Met, %	0.593	0.514
Cys, %	0.239	0.24
Met+Cys, %	0.834	0.75
Thr, %	0.904	0.812
Trp, %	0.306	0.275
Val, %	0.973	0.875

^1^ Supplied the following per kg of diet: 7000 IU of vitamin A (acetate); 500 IU of vitamin D3 (cholecalciferol); 250 IU of vitamin D (25-hydroxicholecalciferol); 45 mg of vitamin E; 1 mg of vitamin K3; 1.5 mg of vitamin B1; 3.5 mg of vitamin B2; 1.75 mg of vitamin B6; 0.03 mg of vitamin B12; 8.5 mg of D-pantothenic acid; 22.5 mg of niacin; 0.1 mg of biotin; 0.75 mg of folacin; 20 mg of Fe (chelate of amino acids); 2.5 mg of Cu (sulfate); 7.5 mg of Cu (chelate of glycine); 0.05 mg of Co (sulfate); 40 mg of Zn (oxide); 12.5 mg Zn (chelate of amino acids); 12.5 mg of Mn (oxide); 7.5 of Mn (chelate of glycine); 0.35 mg of I, 0.5 of Se (organic); and 0.1 mg of Se (inorganic).

**Table 2:** Effect of feeding strategy on the average daily gain (ADG), average daily feed intake (ADFI) and feed conversion ratio (FCR) of small and medium pigs fed using two different feeding management strategies for each period.

**BW Category**	**Strategy ^1^**	**Day0–10**			**Day 10–14**			**Day 14–36**		
		**ADG, g/d**	**ADFI, g/d**	**FCR**	**ADG, g/d**	**ADFI, g/d**	**FCR**	**ADG, g/d**	**ADFI, g/d**	**FCR**
Medium pigs	FIXED	168	178	1.10	163	241	1.50	349	452	1.30
	TBW	163	185	1.11	220	277	1.27	335	446	1.34
SEM ^2^		9.0	8.4	0.025	13.0	11.2	0.085	8.4	11.3	0.021
*p* value ^3^		0.708	0.581	0.907	0.001	0.029	0.069	0.264	0.729	0.215
**BW Category**	**Strategy ^1^**	**Day 0–10**			**Day 10–21**			**Day 21–36**		
		**ADG, g/d**	**ADFI, g/d**	**FCR**	**ADG, g/d**	**ADFI, g/d**	**FCR**	**ADG, g/d**	**ADFI, g/d**	**FCR**
Small pigs	FIXED	141	166	1.17	199	265	1.34	339	488	1.45
	TBW	144	164	1.19	216	296	1.37	332	467	1.41
SEM ^2^		9.2	7.4	0.045	7.4	12.5	0.052	8.4	11.1	0.032
*p* value ^3^		0.798	0.843	0.773	0.118	0.097	0.728	0.590	0.193	0.433

^1^ FIXED: feeding strategy consisting of allowing pigs to consume a pre-starter diet until d10 post-weaning; TBW: feeding strategy consisting of allowing pigs to consume a pre-starter diet until they achieved a BW of 7.9 kg. ^2^ Standard error of the mean. ^3^ *p*-values are derived from the *t*-test.

**Table 3:** Effect of feeding strategy on the average daily gain (ADG), average daily feed intake (ADFI), feed conversion ratio (FCR) and mortality of small and medium pigs fed using two different feeding management strategies for the whole experimental period.

BW Category	Strategy ^1^	Day 0–36			Mortality ^3^, Deaths per Pen		
		ADG, g/d	ADFI, g/d	FCR	Day 0–10	Day 10–36	Day 0–36
Medium pigs	FIXED	277	353	1.27	0.09 ± 0.090 (0.8%)	0.36 ^b^ ± 0.182 (3.5%)	0.46 ± 0.203 (4.1%)
	TBW	276	355	1.29	0 ± 0.000 (0.0%)	0.46 ^b^ ± 0.203 (4.1%)	0.46 ± 0.203 (4.1%)
Small pigs	FIXED	242	330	1.33	0.09 ± 0.090 (0.8%)	0.73 ^a^ ± 0.257 (6.6%)	0.82 ± 0.273 (7.4%)
	TBW	244	330	1.34	0.18 ± 0.129 (1.7%)	0.46 ^b^ ± 0.203 (4.1%)	0.64 ± 0.241 (5.8%)
SEM ^2^		8.1	9.1	0.020	-	-	-
*p* value	Strategy	0.979	0.916	0.984	1.000	0.202	0.193
	BW Category	<0.001	0.014	<0.001	<0.001	0.002	<0.001
	Interaction	0.853	0.903	0.453	<0.001	0.007	0.303

^1^ FIXED: feeding strategy consisting of allowing pigs to consume a pre-starter diet until d10 post-weaning; TBW: feeding strategy consisting of allowing pigs to consume a pre-starter diet until they achieved a BW of 7.9 kg. ^2^ Standard error of the mean. ^3^ Mortality is reported as ‘deaths per pen ± standard error’, with the corresponding percentage provided in parentheses. Values of mortality within a column with different letters significantly differ according to the Tukey post-hoc test (a,b: *p* < 0.05).

**Table 4:** Effect of feeding strategy on the average daily net energy intake (MJ/day; ADEI), average daily digestible lysine intake (dig Lys g/day; ADLysI), net energy conversion efficiency (MJ/kg; NECE) and lysine conversion efficiency (dig Lys g/Kg) of small and medium pigs fed using two different feeding management strategies for the whole experimental period.

**BW Category**	**Strategy**	**Day 0–10**				**Day 10–14**				**Day 14–36**			
		**ADEI**	**NECE**	**ADLysI**	**LysCE**	**ADEI**	**NECE**	**ADLysI**	**LysCE**	**ADEI**	**NECE**	**ADLysI**	**LysCE**
Medium pigs	FIXED	1.9	11.7	3.30	20.4	2.48	15.70	4.32	27.3	4.65	13.30	8.09	23.20
	TBW	1.97	11.8	3.43	20.5	2.94	13.60	5.12	23.6	4.59	13.70	7.98	23.90
SEM		0.094	0.289	0.164	0.502	0.11	0.806	0.191	1.400	0.124	0.222	0.215	0.386
*p* value		0.581	0.907	0.581	0.907	0.007	0.076	0.008	0.075	0.729	0.215	0.729	0.215
**BW Category**	**Strategy**	**Day 0–10**				**Day 10–21**				**Day 21–36**			
		**ADEI**	**NECE**	**ADLysI**	**LysCE**	**ADEI**	**NECE**	**ADLysI**	**LysCE**	**ADEI**	**NECE**	**ADLysI**	**LysCE**
Small pigs	FIXED	1.77	12.5	3.07	21.70	2.72	13.80	4.74	24.10	3.42	10.14	5.96	17.60
	TBW	1.74	12.7	3.03	22.10	3.15	14.60	5.47	25.40	3.27	9.87	5.69	17.20
SEM		0.084	0.508	0.146	0.883	0.132	0.584	0.230	1.010	0.080	0.236	0.138	0.411
*p* value		0.843	0.773	0.843	0.773	0.035	0.365	0.036	0.365	0.193	0.433	0.193	0.433

## Data Availability

The datasets used and analyzed during the current study are available from the corresponding author on reasonable request.

## Supplementary Materials

The following supporting information can be downloaded at: https://www.mdpi.com/article/10.3390/ani14233471/s1, Table S1: Raw data used for the analysis.
